# Association Between *SCN1A* rs2298771, *SCN1A* rs10188577, *SCN2A* rs17183814, and *SCN2A* rs2304016 Polymorphisms and Responsiveness to Antiepileptic Drugs: A Meta-Analysis

**DOI:** 10.3389/fneur.2020.591828

**Published:** 2021-01-14

**Authors:** Mengmeng Li, Rui Zhong, Yingxue Lu, Qian Zhao, Guangjian Li, Weihong Lin

**Affiliations:** Department of Neurology, The First Hospital of Jilin University, Changchun, China

**Keywords:** antiepileptic drugs, responsiveness, resistance, polymorphisms, *SCN1A*, *SCN2A*

## Abstract

**Background:** *SCN1A* and *SCN2A* genes have been reported to be associated with the efficacy of single and combined antiepileptic therapy, but the results remain contradictory. Previous meta-analyses on this topic mainly focused on the *SCN1A* rs3812718 polymorphism. However, meta-analyses focused on *SCN1A* rs2298771, *SCN1A* rs10188577, *SCN2A* rs17183814, or *SCN2A* rs2304016 polymorphisms are scarce or non-existent.

**Objective:** We aimed to conduct a meta-analysis to determine the effects of *SCN1A* rs2298771, *SCN1A* rs10188577, *SCN2A* rs17183814, and *SCN2A* rs2304016 polymorphisms on resistance to antiepileptic drugs (AEDs).

**Methods:** We searched the PubMed, Embase, Cochrane Library, WANFANG, and CNKI databases up to June 2020 to collect studies on the association of *SCN1A* and *SCN2A* polymorphisms with reactivity to AEDs. We calculated the pooled odds ratios (ORs) under the allelic, homozygous, heterozygous, dominant, and recessive genetic models to identify the association between the four single-nucleotide polymorphisms (SNPs) and resistance to AEDs.

**Results:** Our meta-analysis included 19 eligible studies. The results showed that the *SCN1A* rs2298771 polymorphism was related to AED resistance in the allelic, homozygous, and recessive genetic models (G vs. A: OR = 1.20, 95% CI: 1.012–1.424; GG vs. AA: OR = 1.567, 95% CI: 1.147–2.142; GG vs. AA + AG: OR = 1.408, 95% CI: 1.053–1.882). The homozygous model remained significant after Bonferroni correction (*P* < 0.0125). Further subgroup analyses demonstrated the significance of the correlation in the dominant model in Caucasians (South Asians) after Bonferroni correction (GG + GA vs. AA: OR = 1.620, 95% CI: 1.165–2.252). However, no association between *SCN1A* rs2298771 polymorphism and resistance to AEDs was found in Asians or Caucasians (non-South Asians). For *SCN1A* rs10188577, *SCN2A* rs17183814, and *SCN2A* rs2304016 polymorphisms, the correlations with responsiveness to AEDs were not significant in the overall population nor in any subgroup after conducting the Bonferroni correction. The results for *SCN1A* rs2298771, *SCN1A* rs10188577, and *SCN2A* rs2304016 polymorphisms were stable and reliable according to sensitivity analysis and Begg and Egger tests. However, the results for *SCN2A* rs17183814 polymorphism have to be treated cautiously owing to the significant publication bias revealed by Begg and Egger tests.

**Conclusions:** The present meta-analysis indicated that *SCN1A* rs2298771 polymorphism significantly affects resistance to AEDs in the overall population and Caucasians (South Asians). There were no significant correlations between *SCN1A* rs10188577, *SCN2A* rs17183814, and *SCN2A* rs2304016 polymorphisms and resistance to AEDs.

## Introduction

Epilepsy is one of the most common chronic brain diseases, affecting over 70 million people worldwide (1). Antiepileptic drug (AED) therapy is the first-line treatment for patients with epilepsy (PWE); however, one-third of the individuals show drug resistance (2). The underlying mechanism of AED resistance has not been completely elucidated, but genetic factors may be important for the interpersonal differences seen in drug efficacy (3). The current relevant studies mainly focus on polymorphisms of genes that encode metabolic enzymes, transporters, and drug target molecules (4). The voltage-gated sodium (Nav) channel is an important target of AEDs. In the brain, Nav channels consist of a 260-kDa α subunit and four β subunits (β1–β4) of 33–36 kDa; the α subunit is the functional subunit and plays an important role in the generation and propagation of neuronal action potentials (5, 6).

As key genes in the encoding of Nav channels, *SCN1A* and *SCN2A* are associated with the efficacy, dosage, and toxicity of multiple AEDs (3). *SCN1A* rs2298771, *SCN1A* rs10188577, *SCN2A* rs17183814, and *SCN2A* rs2304016 are common single-nucleotide polymorphisms (SNPs) of these genes, and their association with AED resistance has been widely explored. However, the results are still contradictory. For example, Feng et al. (7) found that *SCN1A* rs10188577 polymorphism was related to valproic acid (VPA) resistance in Chinese children with generalized epilepsy (*P* = 0.035). However, another study showed that the association between *SCN1A* rs10188577 polymorphism and response to sodium channel blockers (SCB; including phenytoin, carbamazepine, oxcarbazepine, lamotrigin, topiramate, and valproic acid) was only marginally significant in Caucasians with epilepsy (*P* = 0.049) (8). Moreover, several other studies did not support this correlation (5, 9–11). A similar scenario can be found with *SCN1A* rs2298771, *SCN2A* rs17183814, and *SCN2A* rs2304016 polymorphisms (5, 7, 9–26). In 2013, a meta-analysis performed by Haerian et al. did not find a correlation between *SCN1A* rs2298771 and *SCN2A* rs17183814 polymorphisms and AEDs resistance (10). Another meta-analysis showed that the A allele of the *SCN1A* rs2298771, and the AA genotype in particular, significantly affected the responsiveness to SCB-AEDs (27).

The aforementioned findings are inconsistent. Furthermore, to date, there has been no meta-analysis on the association of *SCN1A* rs10188577 and *SCN2A* rs2304016 polymorphisms with responsiveness to AEDs. Therefore, we conducted a meta-analysis to clarify the association between the aforementioned four gene polymorphisms and AED efficacy.

## Methods

This meta-analysis adheres to the Preferred Reporting Items for Systematic Reviews and Meta-Analyses (PRISMA) guidelines.

### Search Strategy

PubMed, Embase, Cochrane Library, WANFANG, and CNKI databases were searched by two reviewers (M.L. and R.Z.) independently for eligible studies published until June 2020. We used the following search terms: (sodium channel 1.1 OR *SCN1A* OR sodium channel neuronal type I alpha subunit OR *SCN2A* OR sodium channel neuronal type 2 alpha subunit) AND (epilepsy OR seizure) AND [anti-epileptic drug(s) OR antiepileptic drug(s) OR anticonvulsant drugs OR drug-resistant OR resistance OR resistant OR refractory OR response OR drug responsiveness].

### Selection Criteria

The included studies met the following criteria: (1) Design: well-designed cohort or case–control study; (2) Participants: the case group includes individuals who were diagnosed with epilepsy and who showed AED resistance, and the control group includes PWE sensitive to AEDs; (3) Outcome: the original paper describes detailed genotype data including at least one of the target SNPs (*SCN1A* rs2298771, *SCN1A* rs10188577, *SCN2A* rs17183814, or *SCN2A* rs2304016); (4) the diagnosis of epilepsy is in conformity with the International League against Epilepsy (ILAE) guidelines; (5) the genotype distributions of the control group satisfy the Hardy–Weinberg equilibrium (HWE; *P* > 0.01); (6) If studies had duplicate or overlapping data, we chose the largest study. Studies with the following characteristics were excluded: (1) case reports, editorials, comments, reviews, abstracts, or meta-analyses; (2) the control group is composed of healthy people rather than of PWE sensitive to AEDs; (3) articles without complete information.

### Data Extraction and Quality Assessment

Two reviewers independently extracted the data from the studies that met the aforementioned criteria and resolved the discrepancies in data extraction through discussion. Data extraction was as follows: first author, publication year, country, ethnicity, age, gender, types of epilepsy and AED therapy, definition of drug resistance and drug responsiveness, number of cases and controls, genotype and allele frequency, HWE principle, and the Newcastle–Ottawa Scale (NOS) score (28). We used NOS to assess the quality of each included study and defined studies with an NOS score ≥6 as high-quality research.

### Statistical Analysis

We used Stata 12.0 software to perform data analysis. Pooled odds ratios (ORs) and 95% CIs were used to assess the association of *SCN1A* rs2298771, *SCN1A* rs10188577, *SCN2A* rs17183814, and *SCN2A* rs2304016 polymorphisms with responsiveness to AEDs. The combined ORs were determined for the allelic, homozygous, heterozygous, dominant, and recessive genetic models. The statistical significance of the combined ORs was tested using the *Z*-test; *P* < 0.05 was deemed significant. We assessed heterogeneity among the studies using the Cochran Q test and *I*^2^ test. *I*^2^ >50% or *P* < 0.05 was regarded as significant heterogeneity. When heterogeneity was significant, the random-effects model was applied to pool the results; otherwise, the fixed-effects model was applied. Subgroup analyses were performed to assess the correlations in different ethnicities [Asians, Caucasians (South Asians), and Caucasians (non-South Asians)]. To evaluate the stability of our results, we deleted one study at a time sequentially and calculated the combined ORs of the remaining studies. In addition, we evaluated publication bias by drawing a funnel plot, and further confirmed it using Begg or Egger tests; *P* < 0.05 was defined as significant publication bias. Finally, the Bonferroni method was applied for multiple comparisons. We evaluated four SNPs in this meta-analysis; thus, *P* < 0.0125 after Bonferroni correction was considered significant (29, 30).

## Results

### Study Inclusion and Characteristics

**Figure 1:**
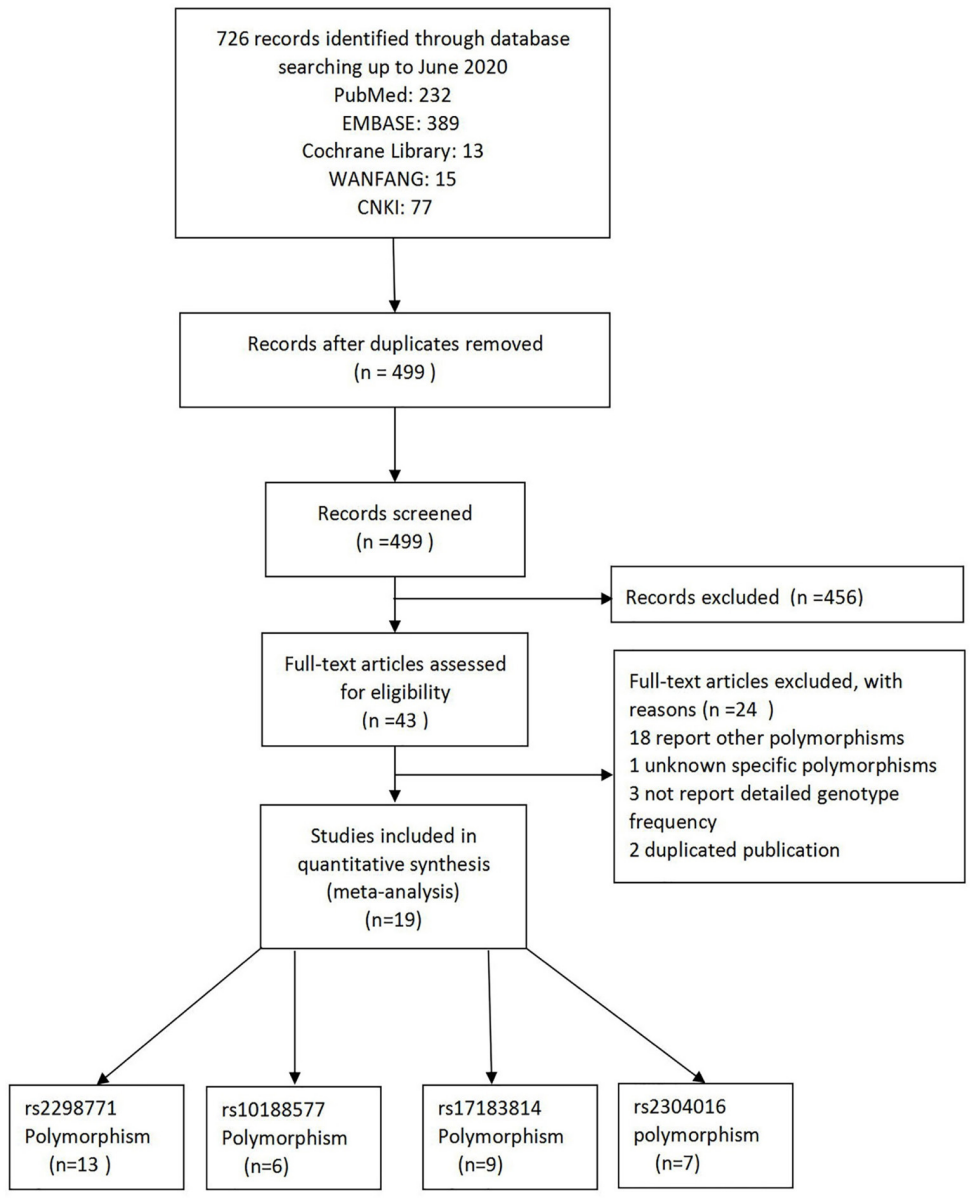
Flow diagram of literature screening.

As a result of our retrieval strategy, a total of 726 records were identified. After eliminating 227 duplicate studies, we screened the titles and abstracts of the remaining 499 articles. A total of 456 studies were excluded based on the analysis of titles and abstracts. Then, full-text appraisal was performed for the remaining 43 articles, and 24 articles were excluded for different reasons. Of these, 18 articles reported other polymorphisms, 1 article did not explain specific polymorphisms, 3 articles did not provide detailed genotype frequency, and 2 articles had the same research data. Finally, 19 studies were included in our meta-analysis (5, 7–24). The screening process is shown in Figure 1. In addition to the one study that did not conform to the HWE principle, a total of 13 studies (18 groups) analyzed the *SCN1A* rs2298771 polymorphism; this represented 2,368 drug-resistant and 2,665 drug-responsive PWE. *SCN1A* rs10188577 was studied in 6 articles (10 groups), involving 1,860 drug-resistant and 1,790 drug-responsive PWE. Nine studies (14 groups) analyzed the *SCN2A* rs17183814 polymorphism, representing 1,973 drug-resistant and 2,172 drug-responsive PWE. Excluding one study that did not agree with the HWE principle, 7 studies (12 groups) assessed the *SCN2A* rs2304016 polymorphism; they consisted of 1,797 drug-resistant and 1,947 drug-responsive PWE. According to the Newcastle–Ottawa scale, the quality score of each included study was ≥6, indicating good quality overall. Table 1 displays the main characteristics and quality assessment results of the eligible studies. Table 2 shows *SCN1A* rs2298771, *SCN1A* rs10188577, *SCN2A* rs17183814, and *SCN2A* rs2304016 genotypes and allele distributions among drug non-responders and responders with epilepsy.

**Table 1:** Characteristics of studies on *SCN1A* and *SCN2A* polymorphisms and resistance to AEDs included in the meta-analysis.

**First author**	**Year**	**Country**	**Ethnicity**	**Age(years)**	**Male/Female**	**Drugs**	**Types of epilepsy**	**SCN family gene loci**	**NR definition**	**R definition**	**NR**	**R**	**Quality**
Kwan, P.(G1)	2008	Chinese Han	Asians	36.3 ± 14.5	239/232	AEDs	Various	1,2,3,4	≥1 seizure per month on average with ≥2 AEDs	≥ 1 year seizure-free	199	272	8
Kwan, P.(G2)	2008	Chinese Han	Asians	>15	unclear	PHT, CBZ, OXC, LTG, and TPM	Various	1,2,3,4	≥1 seizure per month on average with ≥2 SCB-AEDs	≥ 1 year seizure-free	200	177	8
Lakhan, R.	2009	Northern Indian	Caucasians (South Asians)	24.2 ± 11.3	242/94	AEDs	Various	1,3	≥4 seizures of 1 year with 3 AEDs; After epilepsy surgery	≥ 1 year seizure-free	117	219	7
Sánchez, M. B.	2010	Spanish	Caucasians (non-South Asians)	NR:27.0 ± 18.5; R: 26.0 ± 19.8	144/145	AEDs	Various	1	≥4 seizures of 1 year with >3 AEDs	≥ 1 year seizure-free	111	178	8
Boting Zhou	2012	Chinese Han	Asians	Male:30.5 ± 17.6; Female:30.7 ± 16.8	137/135	CBZ	Focal	1	No seizure free	≥ 1 year seizure-free	123	149	6
Haerian, B.S.(G1)	2013	Malaysian	Asians	NR: 30 ± 15; R:28 ± 15	134/117	CBZ or VPA	Various	1,2,3,4	No seizure free; After epilepsy surgery	≥ 1 year seizure-free	125	126	7
Haerian, B.S.(G2)	2013	Indian	Caucasians (South Asians)	NR:33 ± 17; R:30 ± 16	87/75	CBZ or VPA	Various	1,2,3,4	No seizure free; After epilepsy surgery	≥ 1 year seizure-free	70	92	7
Haerian, B.S.(G3)	2013	Northern Chinese	Asians	NR:33 ± 17; R: 34 ± 19	160/128	CBZ or VPA	Various	1,2,3,4	No seizure free; After epilepsy surgery	≥ 1 year seizure-free	140	148	7
Haerian, B.S.(G4)	2013	Chinese (Hong Kong)	Asians	NR:38 ±14; R: 38 ± 16	405/398	AEDs	Various	1,2,3,4	≥1 seizure per month with ≥2 AEDs	≥ 1 year seizure-free	467	336	7
T S C Yip	2013	Australian	Caucasians (non-South Asians)	1–86 (median age:27)	247/272	PHT, CBZ, OXC, LTG, VPA and TPM	Various	2	≥4 seizures of 1 year with ≥2 SCB-AEDs	≥ 1 year seizure-free	358	161	6
Yuze Cao	2013	Chinese Han	Asians	NR: 19.0 ± 9.8; R: 18.2 ± 16.8	298/182	AEDs	Various	3,4	No seizure free with ≥2 AEDs	Seizure free for ≥3 times of the longest interval of seizure (at least ≥ 1 year)	207	273	7
Chunlai Ma(G1)	2014	Chinese Han	Asians	unclear	unclear	CBZ or OXC	Various	1,3,4	≥4 seizures of 1 year	Seizure free for ≥3 times of the longest interval of seizure (at least ≥ 1 year)	156	170	6
Chunlai Ma(G2)	2014	Chinese Han	Asians	unclear	unclear	CBZ+OXC	Various	1,3,4	≥4 seizures of 1 year	Seizure free for ≥3 times of the longest interval of seizure (at least ≥ 1 year)	90	37	6
Ping Wang	2014	Chinese Han	Asians	Male:25.2 ± 11.6; Female:26.5 ± 12.9	220/131	CBZ	Focal	1	No seizure free	≥ 1 year seizure-free	157	194	6
Xi Wu	2014	Chinese	Asians	≤ 14: 139 >14:62	136/65	VPA	Various	4	No seizure free of 1 year	≥ 1 year seizure-free	70	131	7
Luo Zhou	2015	Chinese Han	Asians	NR:22.98 ± 11.21; R: 22.36 ± 14.10	247/144	AEDs	Various	4	No seizure free with ≥2 AEDs	Seizure free for ≥3 times of the longest interval of seizure (at least ≥ 1 year)	156	235	7
Daci, A.	2015	Kosovar	Caucasians (non-South Asians)	32.9 ± 15.5	82/63	CBZ	Not reported	1	≥4 seizures of 1 year	≥ 1 year seizure-free	46	99	7
Abo El Fotoh, W.M.	2016	Egyptian	Caucasians (non-South Asians)	NR: 7.43 ± 2.95 R: 8.34 ± 2.95	80/50	AEDs	Various	1	Uncontrolled seizures over 1 year with ≥3 AEDs	≥ 1 year seizure-free	50	80	7
Baghel, R.	2016	North Indian	Caucasians (South Asians)	> 5	214/165	CBZ or VPA or PB or PHT monotherapy	Various	1, 2	≥1 seizure in last 10 months	≥ 10 months seizure-free	154	225	6
Bertok, S.	2017	Slovenian	Caucasians (non-South Asians)	children and adolescents	91/125	AEDs	Various	1	Uncontrolled seizures over 1 year with ≥2 AEDs	≥ 1 year seizure-free	102	114	7
Weixing Feng	2018	Chinese	Asians	NR: 8.21 ± 4.56 R: 9.39 ± 4.07	107/67	VPA or VPA+AEDs	Generalized	1,2,3,4	≤ 50% seizure reduction	>50% seizure reduction	33	141	6
Nazish, H. R.	2018	Pakistani	Caucasians (South Asians)	18.7 ± 9.5	39/41	CBZ	Various	1,3	Poor seizure-controlled at 6th month of CBZ therapy	Well seizure-controlled at 6th month of CBZ therapy	44	36	6
Pejanovic- Skobic, N.	2019	Bosnia and Herzegovina	Caucasians (non-South Asians)	NR: 38.76 ± 14.61 R: 35.24 ± 15.62	48/52	LTG	Focal	3	≥1 seizure of 1 year	≥ 1 year seizure-free	33	67	7
Lihong Shi	2019	Chinese Han	Asians	NR: 3.8–32.7 R: 3.5–14.4	159/94	VPA	Various	1,2,3,4	≥4 seizures of 1 year	≥ 1 year seizure-free	128	125	6

*AEDs, antiepileptic drugs; CBZ, carbamazepine; PHT, phenytoin; OXC, oxcarbazepine; LTG, lamotrigin; TPM, topiramate; VPA, valproic acid; PB, phenobarbital; SCB-AEDs, sodium channel blocking AEDs (including PHT, CBZ, OXC, LTG, TPM and VPA); NR, drug non-responders; R, drug responders; 1, rs2298771; 2, rs10188577; 3, rs17183814; 4, rs2304016*.

**Table 2:** Genotype and allele distributions of *SCN1A* and *SCN2A* gene loci.

**SNPs (W > M)**	**First author**	**NR WW**	**NR WM**	**NR MM**	**NR W**	**NR M**	**R WW**	**R WM**	**R MM**	**R W**	**R M**	**HWE(p)**
rs2298771(A > G)	Kwan, P.(G1)	163	32	3	358	38	223	48	0	494	48	>0.05
	Kwan, P.(G2)	162	34	3	358	40	144	32	0	320	32	>0.05
	Lakhan, R.	50	62	5	162	72	94	117	8	305	133	<0.01
	Sánchez, M. B.	43	54	14	140	82	71	88	19	230	126	>0.05
	Boting Zhou	83	36	4	202	44	121	28	0	270	28	>0.05
	Haerian, B.S.(G1)	110	23	1	243	25	103	29	3	235	35	>0.05
	Haerian, B.S.(G2)	41	22	1	104	24	58	24	3	140	30	>0.05
	Haerian, B.S.(G3)	74	37	5	185	47	86	30	3	202	36	>0.05
	Haerian, B.S.(G4)	386	72	5	844	82	272	58	2	602	62	>0.05
	Chunlai Ma(G1)	124	29	3	277	35	133	36	1	302	38	>0.05
	Chunlai Ma(G2)	72	18	0	162	18	34	3	0	71	3	>0.05
	Ping Wang	105	50	2	260	54	157	35	2	349	39	>0.05
	Daci, A.	8	27	11	43	49	17	56	26	90	108	>0.05
	Abo El Fotoh, W.M.	14	29	7	57	43	41	28	11	110	50	>0.05
	Baghel, R.	74	63	17	211	97	132	76	17	340	110	>0.05
	Bertok, S.	54	37	11	145	59	41	62	11	144	84	>0.05
	Weixing Feng	28	5	0	61	5	117	22	1	256	24	>0.05
	Nazish, H. R.	10	18	16	38	50	19	12	5	50	22	>0.05
	Lihong Shi	107	19	2	233	23	103	20	2	226	24	>0.05
rs10188577(T>C)	Kwan, P.(G1)	127	65	5	319	75	164	94	13	422	120	>0.05
	Kwan, P.(G2)	129	64	5	322	74	107	59	10	273	79	>0.05
	Haerian, B.S.(G1)	88	41	7	217	55	89	56	3	234	62	>0.05
	Haerian, B.S.(G2)	37	24	8	98	40	45	35	7	125	49	>0.05
	Haerian, B.S.(G3)	74	47	4	195	55	71	41	12	183	65	>0.05
	Haerian, B.S.(G4)	303	142	17	748	176	205	114	13	524	140	>0.05
	T S C Yip	151	173	34	475	241	80	57	24	217	105	>0.01
	Baghel, R.	83	61	10	227	81	113	93	19	319	131	>0.05
	Weixing Feng	25	8	0	58	8	82	51	8	215	67	>0.05
	Lihong Shi	83	40	5	206	50	85	38	2	208	42	>0.05
rs17183814(G > A)	Kwan, P.(G1)	141	49	7	331	63	202	62	5	466	72	>0.05
	Kwan, P.(G2)	143	48	7	334	62	133	39	4	305	47	>0.05
	Lakhan, R.	83	29	5	195	39	174	42	3	390	48	>0.05
	Haerian, B.S.(G1)	98	33	1	229	35	96	33	3	225	39	>0.05
	Haerian, B.S.(G2)	49	18	0	116	18	56	24	6	136	36	>0.05
	Haerian, B.S.(G3)	108	10	1	226	12	91	23	2	205	27	>0.05
	Haerian, B.S.(G4)	320	119	13	759	145	246	75	5	567	85	>0.05
	Yuze Cao	141	54	12	336	78	193	73	7	459	87	>0.05
	Chunlai Ma(G1)	119	35	2	273	39	125	41	4	291	49	>0.05
	Chunlai Ma(G2)	72	17	1	161	19	27	9	1	63	11	>0.05
	Weixing Feng	26	6	1	58	8	102	35	3	239	41	>0.05
	Nazish, H. R.	10	18	16	38	50	18	11	7	47	25	>0.05
	Pejanovic-Skobic, N	30	3	0	63	3	57	10	0	124	10	>0.05
	Lihong Shi	99	27	2	225	31	100	23	2	223	27	>0.05
rs2304016(A > G)	Kwan, P.(G1)	181	14	3	376	20	216	51	1	483	53	>0.05
	Kwan, P.(G2)	180	16	3	376	22	142	33	1	317	35	>0.05
	Haerian, B.S.(G1)	114	14	1	242	16	123	11	0	257	11	>0.05
	Haerian, B.S.(G2)	66	0	0	132	0	84	2	0	170	2	>0.05
	Haerian, B.S.(G3)	111	4	0	226	4	113	5	0	231	5	>0.05
	Haerian, B.S.(G4)	410	44	3	864	50	267	59	2	593	63	>0.05
	Yuze Cao	133	49	25	315	99	187	65	21	439	107	<0.01
	Chunlai Ma(G1)	121	32	3	274	38	128	40	2	296	44	>0.05
	Chunlai Ma(G2)	76	13	1	165	15	29	8	0	66	8	>0.05
	Xi Wu	63	7	0	133	7	95	35	0	225	35	>0.05
	Luo Zhou	127	28	1	282	30	188	46	1	422	48	>0.05
	Weixing Feng	26	7	0	59	7	108	28	4	244	36	>0.05
	Lihong Shi	101	26	1	228	28	109	14	2	232	18	>0.05

*NR, drug non-responders; R, drug responders; W, wild-type; M, mutant-type; HWE, Hardy–Weinberg equilibrium*.

### Association Between the *SCN1A* rs2298771 Polymorphism and Resistance to AEDs

**Figure 2:**
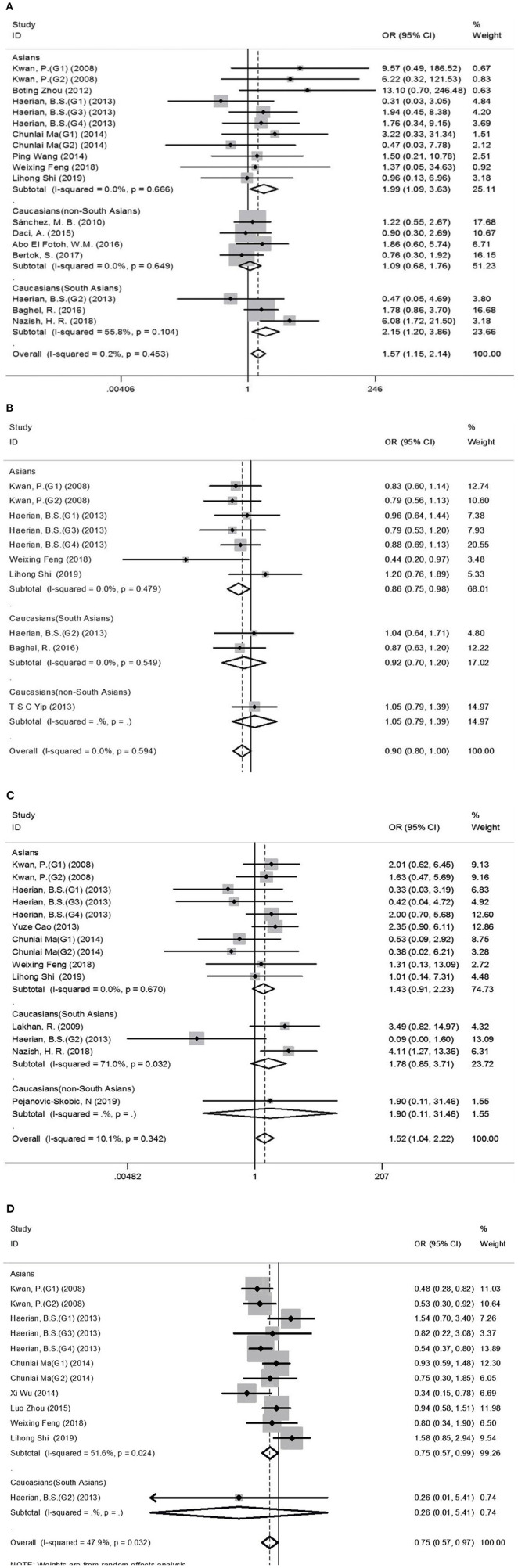
Forest plots for the association between the *SCN1A* rs2298771, *SCN1A* rs10188577, *SCN2A* rs17183814, and *SCN2A* rs2304016 polymorphisms and resistance to antiepileptic drugs. **(A)**, *SCN1A* rs2298771 polymorphism (GG vs. AA). **(B)** *SCN1A*rs10188577 polymorphism (C vs. T). **(C)**, *SCN2A* rs17183814 polymorphism (AA vs. GG). **(D)** *SCN2A* rs2304016 polymorphism (G vs. A). The sizes of the squares reflect the study's weight and horizontal lines represent 95% CI; the center of diamonds reflects the overall odds ratio (OR), and the horizontal span of the diamond represents the 95% CI of the OR.

**Figure 3:**
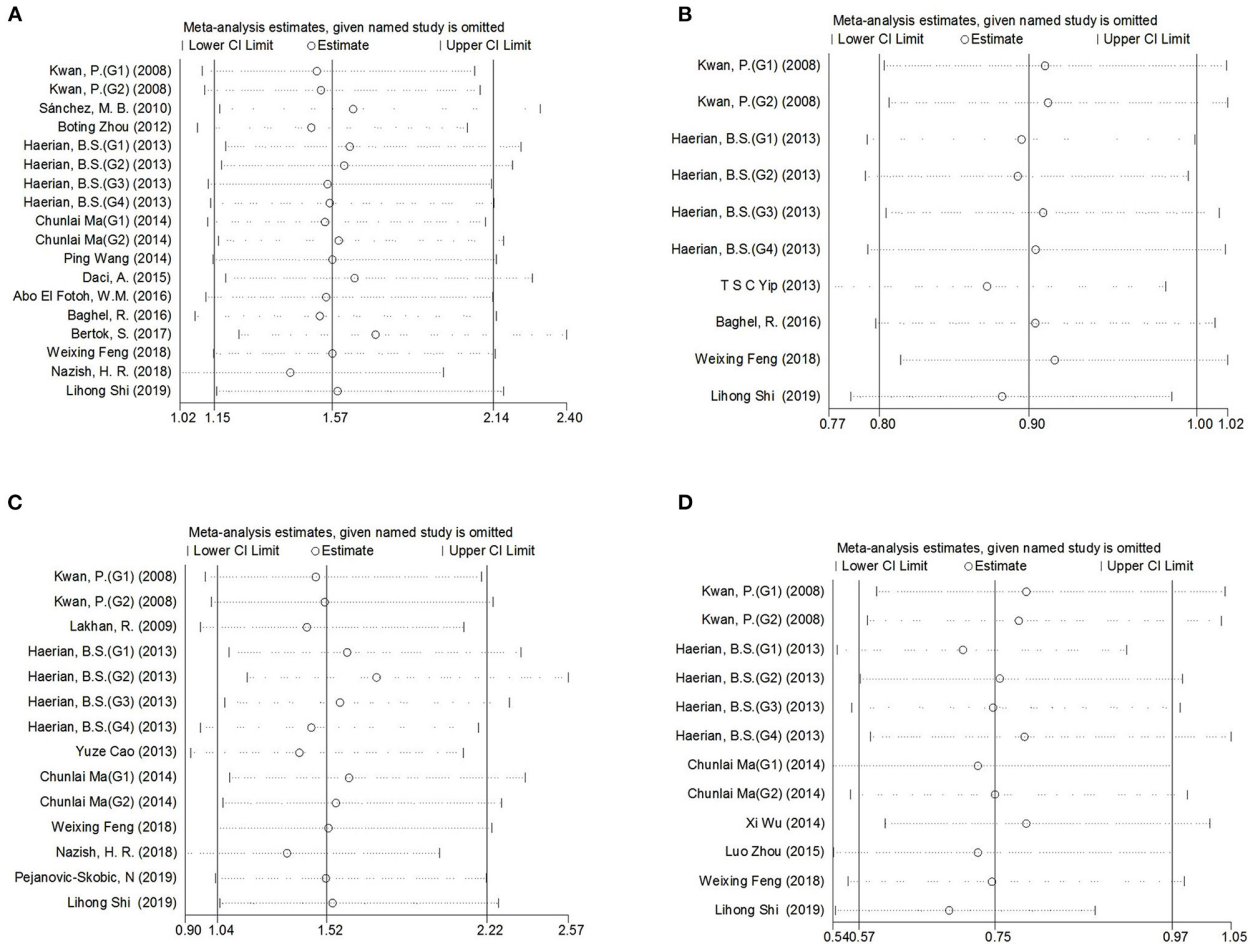
Sensitivity analysis indicated the stability of the results. **(A)** *SCN1A* rs2298771 polymorphism (GG vs. AA). **(B)** *SCN1A* rs10188577 polymorphism (C vs. T). **(C)**, *SCN2A* rs17183814 polymorphism (AA vs. GG). **(D)**, *SCN2A* rs2304016 polymorphism (G vs. A).

**Figure 4:**
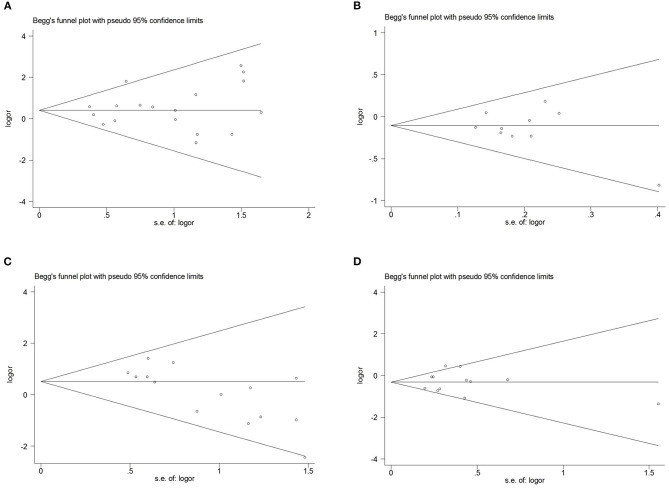
Begg's funnel plot for publication bias test. **(A)**, *SCN1A* rs2298771 polymorphism (GG vs. AA). **(B)**, *SCN1A* rs10188577 polymorphism (C vs. T). **(C)**, *SCN2A* rs17183814 polymorphism (AA vs. GG). **(D)** *SCN2A* rs2304016 polymorphism (G vs. A).

Comprehensive analysis showed a significant correlation between the *SCN1A* rs2298771 polymorphism and resistance to AEDs under the allelic, homozygous, and recessive genetic models (G vs. A: OR = 1.20, 95% CI: 1.012–1.424, *P* = 0.036, *I*^2^ = 53.2%; GG vs. AA: OR = 1.567, 95% CI: 1.147–2.142, *P* = 0.005, *I*^2^ = 0.2%; GG vs. AA + AG: OR = 1.408, 95% CI: 1.053–1.882, *P* = 0.021, *I*^2^ = 0.0%). Further subgroup analyses reported similar results among the Asians (G vs. A: OR = 1.184, 95% CI: 1.018–1.377, *P* = 0.028, *I*^2^ = 44.6%; GG vs. AA: OR = 1.988, 95% CI: 1.090–3.629, *P* = 0.025, *I*^2^ = 0.0%; GG vs. AA + AG: OR = 1.914, 95% CI: 1.050–3.490, *P* = 0.034, *I*^2^ = 0.0%). In addition, the correlation was also significant in Caucasians (South Asians) in the heterozygous, dominant, and recessive models (GA vs. AA: OR=1.541, 95% CI: 1.086–2.186, *P* = 0.015, *I*^2^ = 0.0%; GG + GA vs. AA: OR = 1.620, 95% CI: 1.165–2.252, *P* = 0.004, *I*^2^ = 47.4%; GG vs. AA + AG: OR = 1.755, 95% CI: 1.003–3.071, *P* = 0.049, *I*^2^ = 35.3%). However, no significant associations were found in Caucasians (non-South Asians). After Bonferroni correction, the associations between the *SCN1A* rs2298771 polymorphism and resistance to AEDs only remained significant in the overall population under the homozygous model and in Caucasians (South Asians) under the dominant model. Table 3 and Figure 2A display the results of the *SCN1A* rs2298771 polymorphism. The sensitivity analysis showed stable results (Figure 3A). Begg and Egger tests were performed to evaluate publication bias and indicated its absence (Table 3 and Figure 4A).

**Table 3:** Summary of the association between *SCN1A* and *SCN2A* polymorphisms and resistance to AEDs.

**SNPs**	**Comparisons**	**Ethnicity**	**Number of groups**	**Test of association**		**Model**	**Test of heterogeneity**		**Publication bias**	
				**OR (95%CI)**	***P***		***P***	**I^**2**^(%)**	**Begg**	**Egger**
rs2298771	G vs. A	Overall	18	1.20 (1.012, 1.424)	**0.036**	R	0.004	53.2%	0.495	0.422
		Asians	11	1.184 (1.018, 1.377)	**0.028**	F	0.054	44.6%		
		Caucasians (non-South Asians)	4	1.020 (0.734, 1.419)	0.906	R	0.075	56.5%		
		Caucasians (South Asians)	3	1.603 (0.977, 2.629)	0.062	R	0.062	64.0%		
	GG vs. AA	Overall	18	1.567 (1.147, 2.142)	0.005*	F	0.453	0.2%	0.649	0.496
		Asians	11	1.988 (1.090, 3.629)	**0.025**	F	0.666	0.0%		
		Caucasians (non-South Asians)	4	1.091 (0.677, 1.759)	0.721	F	0.649	0.0%		
		Caucasians (South Asians)	3	2.166 (0.705, 6.648)	0.177	R	0.104	55.8%		
	GA vs. AA	Overall	18	1.173 (0.943, 1.458)	0.152	R	0.002	55.8%	0.289	0.257
		Asians	11	1.119 (0.946, 1.325)	0.190	F	0.054	44.7%		
		Caucasians (non-South Asians)	4	1.058 (0.500, 2.238)	0.883	R	0.002	79.4%		
		Caucasians (South Asians)	3	1.541 (1.086, 2.186)	**0.015**	F	0.458	0.0%		
	GG+GA vs. AA	Overall	18	1.215 (0.978, 1.510)	0.078	R	0.001	58.3%	0.256	0.299
		Asians	11	1.174 (0.924, 1.491)	0.189	R	0.041	47.2%		
		Caucasians (non-South Asians)	4	1.055 (0.539, 2.065)	0.876	R	0.005	76.6%		
		Caucasians (South Asians)	3	1.620 (1.165, 2.252)	0.004*	F	0.149	47.4%		
	GG vs. AA+AG	Overall	18	1.408 (1.053, 1.882)	**0.021**	F	0.71	0.0%	0.596	0.373
		Asians	11	1.914 (1.050, 3.490)	**0.034**	F	0.674	0.0%		
		Caucasians (non-South Asians)	4	1.061 (0.696, 1.617)	0.783	F	0.952	0.0%		
		Caucasians (South Asians)	3	1.755 (1.003, 3.071)	**0.049**	F	0.213	35.3%		
rs10188577	C vs. T	Overall	10	0.897 (0.801, 1.003)	0.057	F	0.594	0.0%	0.721	0.379
		Asians	7	0.858 (0.747, 0.985)	**0.030**	F	0.479	0.0%		
		Caucasians (South Asians)	2	0.917 (0.699, 1.204)	0.534	F	0.549	0.0%		
	CC vs. TT	Overall	10	0.749 (0.556, 1.011)	0.059	F	0.298	15.8%	1	0.961
		Asians	7	0.695 (0.461, 1.048)	0.083	F	0.151	36.3%		
		Caucasians (South Asians)	2	0.902 (0.472, 1.726)	0.756	F	0.344	0.0%		
	CT vs. TT	Overall	10	0.945 (0.819, 1.090)	0.436	F	0.285	17.2%	0.721	0.543
		Asians	7	0.874 (0.739, 1.035)	0.118	F	0.778	0.0%		
		Caucasians (South Asians)	2	0.876 (0.609, 1.259)	0.473	F	0.867	0.0%		
	CC+CT vs. TT	Overall	10	0.912 (0.796, 1.046)	0.188	F	0.478	0.0%	0.858	0.420
		Asians	7	0.853 (0.724, 1.003)	0.055	F	0.731	0.0%		
		Caucasians (South Asians)	2	0.881 (0.625, 1.244)	0.473	F	0.854	0.0%		
	CC vs. TT+TC	Overall	10	0.733 (0.547, 0.982)	**0.038**	F	0.205	25.9%	0.858	0.659
		Asians	7	0.725 (0.483, 1.089)	0.121	F	0.128	39.6%		
		Caucasians (South Asians)	2	0.958 (0.511, 1.797)	0.894	F	0.311	2.7%		
rs17183814	A vs. G	Overall	14	1.050 (0.861, 1.280)	0.629	R	0.011	52.8%	0.080	0.079
		Asians	10	1.070 (0.932, 1.228)	0.337	F	0.135	34.2%		
		Caucasians (South Asians)	3	1.335 (0.617, 2.889)	0.463	R	0.004	81.9%		
	AA vs. GG	Overall	14	1.519 (1.040, 2.219)	**0.030**	F	0.342	10.1%	0.012	0.003
		Asians	10	1.429 (0.914, 2.234)	0.117	F	0.670	0.0%		
		Caucasians (South Asians)	3	1.674 (0.273, 10.261)	0.578	R	0.032	71.0%		
	AG vs. GG	Overall	14	1.046 (0.902, 1.214)	0.550	F	0.257	18.0%	0.125	0.211
		Asians	10	1.012 (0.861, 1.190)	0.882	F	0.371	7.7%		
		Caucasians (South Asians)	3	1.358 (0.913, 2.022)	0.131	F	0.165	44.5%		
	AA+AG vs. GG	Overall	14	1.079 (0.936, 1.245)	0.296	F	0.056	40.8%	0.155	0.183
		Asians	10	1.044 (0.893, 1.219)	0.591	F	0.226	23.6%		
		Caucasians (South Asians)	3	1.470 (0.666, 3.244)	0.340	R	0.024	73.3%		
	AA vs. GG+GA	Overall	14	1.443 (0.993, 2.097)	0.054	F	0.547	0.0%	0.009	0.002
		Asians	10	1.419 (0.908, 2.215)	0.124	F	0.723	0.0%		
		Caucasians (South Asians)	3	1.444 (0.310, 6.731)	0.640	R	0.065	63.3%		
rs2304016	G vs. A	Overall	12	0.745 (0.570, 0.974)	**0.032**	R	0.032	47.9%	0.945	0.915
		Asians	11	0.752 (0.572, 0.988)	**0.040**	R	0.024	51.6%		
	GG vs. AA	Overall	12	1.366 (0.695, 2.681)	0.365	F	0.996	0.0%	0.732	0.874
		Asians	11	1.371 (0.684, 2.749)	0.373	F	0.991	0.0%		
	GA vs. AA	Overall	12	0.679 (0.480, 0.961)	**0.029**	R	0.002	62.1%	0.837	0.824
		Asians	11	0.687 (0.482, 0.980)	**0.038**	R	0.001	65.1%		
	GG+GA vs. AA	Overall	12	0.702 (0.510, 0.964)	**0.029**	R	0.007	57.1%	1	0.833
		Asians	11	0.709 (0.513, 0.981)	**0.038**	R	0.005	60.4%		
	GG vs. AA+AG	Overall	12	1.438 (0.735, 2.814)	0.288	F	0.992	0.0%	0.732	0.823
		Asians	11	1.447 (0.725, 2.889)	0.295	F	0.983	0.0%		

*R, random-effects model; F, fixed-effects model. In the case of the test of association-bold values without asterisk indicate statistically significant and bold values with asterisk indicate significant after Bonferroni correction (p < 0.0125)*.

### Association Between the *SCN1A* rs10188577 Polymorphism and Resistance to AEDs

In the recessive genetic model, the CC genotype of the *SCN1A* rs10188577 was significantly associated with responsiveness to AEDs (OR = 0.733, 95% CI: 0.547–0.982, *P* = 0.038, *I*^2^ = 25.9%). Subgroup analyses indicated that the C allele was associated with sensitivity to AEDs in Asians with epilepsy in the allele model (OR = 0.858, 95% CI: 0.747–0.985, *P* = 0.030, *I*^2^ = 0.0%). However, after Bonferroni correction, there were no significant correlations between the *SCN1A* rs10188577 polymorphism and sensitivity to AEDs. These results are shown in Table 3 and Figure 2B. The results were stable and credible, as proved by the sensitivity analysis (Figure 3B). We did not detect any publication bias (Table 3 and Figure 4B).

### Association Between the *SCN2A* rs17183814 Polymorphism and Resistance to AEDs

In the homozygous genetic model of the *SCN2A* rs17183814 polymorphism, the pooled OR value was 1.519 (95% CI: 1.040–2.219, *P* = 0.030) under the fixed-effects model. However, this correlation was not significant after Bonferroni correction. No association between the *SCN2A* rs17183814 polymorphism and resistance to AEDs was observed in the other four models or in further subgroup analyses. We found no significant heterogeneity (*I*^2^=10.1%; Table 3 and Figure 2C). The sensitivity analysis indicated stable results (Figure 3C). However, publication bias was observed according to Begg and Egger tests (Table 3 and Figure 4C). Therefore, these results should be applied cautiously.

### Association Between the *SCN2A* rs2304016 Polymorphism and Resistance to AEDs

There was a significant correlation between the *SCN2A* rs2304016 polymorphism and AEDs response in the allelic, heterozygous, and dominant genetic models (G vs. A: OR = 0.745, 95% CI: 0.570–0.974, *P* = 0.032, *I*^2^ = 47.9%; GA vs. AA: OR = 0.679, 95% CI: 0.480–0.961, P = 0.029, *I*^2^ = 62.1%; GG + GA vs. AA: OR = 0.702, 95% CI: 0.510–0.964, *P* = 0.029, *I*^2^ = 57.1%). The subgroup analysis in Asians revealed similar results (G vs. A: OR = 0.752, 95% CI: 0.572–0.988, *P* = 0.040, *I*^2^ = 51.6%; GA vs. AA: OR = 0.687, 95% CI: 0.482–0.980, *P* = 0.038, *I*^2^ = 65.1%; GG + GA vs. AA: OR = 0.709, 95% CI: 0.513–0.981, *P* = 0.038, *I*^2^ = 60.4%; Table 3 and Figure 2D). After Bonferroni correction, the aforementioned correlation was not significant. The sensitivity analysis indicated stable results (Figure 3D). There was no significant publication bias (Table 3 and Figure 4D).

## Discussion

The results of our meta-analysis showed that the G allele and the GG genotype of *SCN1A* rs2298771 increase the risk of resistance to AEDs for the overall population with epilepsy and for Asians with epilepsy. In addition, the AA genotype of *SCN1A* rs2298771 implies sensitivity to AEDs for Caucasians (South Asians). Regarding *SCN1A* rs10188577, the CC genotype for the overall population and the C allele for Asians reduce the risk of resistance to AEDs. The A allele and the AA genotype of *SCN2A* rs2304016 were more common in the overall population and Asians with drug-resistant epilepsy. The aforementioned results were stable and reliable after sensitivity analysis and Begg and Egger tests. Moreover, we found that the *SCN2A* rs17183814 polymorphism was related to resistance to AEDs under the homozygous genetic model, but there was significant publication bias, as shown by Begg and Egger tests; thus, this result was unreliable. Unfortunately, after Bonferroni correction, we found that only the AA genotype of *SCN1A* rs2298771 was associated with responsiveness to AEDs in all people and in Caucasians (South Asians), but the correlations between the other three SNPs and resistance to AEDs did not remain significant.

Epilepsy is known to be an ion channel disease. Nav channels are the main targets of many first-line AEDs (31–34). *SCN1A* and *SCN2A* genetic variants may change the response to AEDs (25). *SCN1A* rs2298771 (c.3184A>G/p.Thr1067Ala), located in the *SCN1A* exon region, is an A-to-G variant, which causes the substitution of alanine for threonine (27). Similarly, *SCN2A* rs17183814 (c.56G>A/p.Arg19Lys), situated in the *SCN2A* exon region, is a G-to-A SNP, which generates a conversion of arginine to lysine (12). These two SNPs may affect the structural and functional characteristics of Nav channels and further influence the therapeutic effects of AEDs (27). *SCN1A* rs10188577 (T>C) and *SCN2A* rs2304016 (IVS7-32A>G) are common SNPs of the intron region and are considered to be significant in the regulation of *SCN1A* and *SCN2A* expression, respectively (35).

The relationship between *SCN1A* and *SCN2A* polymorphisms and resistance to AEDs had been extensively studied, but the conclusions were inconsistent. Kwan et al. (9) found that the *SCN1A* rs2298771 polymorphism did not influence the response to AEDs. This finding was confirmed by later studies (5, 7, 10–15). However, some studies found that the *SCN1A* rs2298771 polymorphism was related to AED resistance (16–20). For example, E.F.W. Abo et al. reported that the AG genotype and the G allele frequency of *SCN1A* rs2298771 were significantly higher in AED-resistant patients than in drug responders (18). The *SCN1A* rs10188577 polymorphism was associated with AED resistance in studies performed by Feng et al. (7) and Yip et al. (8). However, several other studies did not support the aforementioned findings (5, 9–11). Lakhan et al. (12) found that A variant of *SCN2A* rs17183814 was more common in patients who were resistant to multiple AEDs. The study conducted by Nazish et al. (20) demonstrated that the GA and AA genotypes of rs17183814 were related to carbamazepine resistance. However, some studies did not find a similar relationship (5, 7, 9, 10, 14, 21, 24). Kwan et al. (9) and Li et al. (26) reported that the A allele and the AA genotype of *SCN2A* rs2304016 were risk factors for AED resistance. In contrast, Shi et al. (5) found that the rs2304016 G allele increased the risk of VPA resistance. Moreover, some studies have shown that the rs2304016 polymorphism does not affect the risk of AED resistance (7, 10, 14, 21–23, 25).

Recently, new case–control studies and cohort studies have been published. Hence, we performed an updated meta-analysis of the relationship between *SCN1A* rs2298771 and *SCN2A* rs17183814 polymorphisms and AED resistance. Furthermore, the study quantitatively evaluated the significance of *SCN1A* rs10188577 and *SCN2A* rs2304016 polymorphisms in AED resistance for the first time. The meta-analysis conducted by Haerian et al. did not find an association of *SCN1A* rs2298771 and *SCN2A* rs17183814 polymorphisms with response to AEDs (10). Our study, similar to this meta-analysis, did not limit the types of AEDs; however, we incorporated new studies (5, 7, 9, 11, 14–21, 24). Therefore, the conclusions of these two meta-analyses were not identical. Bao et al. (27) also performed a meta-analysis. However, they excluded studies that were not limited to SCB-AEDs; this did not happen in our study. Fortunately, their conclusions were similar to ours in that the AA genotype of *SCN1A* rs2298771 predisposed to responsiveness to AEDs. Thus, we speculate that the *SCN1A* rs2298771 polymorphism is related to resistance to multiple AEDs and not merely to SCB-AEDs.

There were certain limitations in our meta-analysis that should be considered while interpreting the results. First, the definition of treatment outcomes was different across the included studies, resulting in heterogeneity in grouping standards. Second, multivariate analysis to adjust for confounding factors, such as age, sex, type/duration/severity of epilepsy, and AEDs type, was not performed in the present meta-analysis because of the limited information offered by the included studies. Third, the Chinese were predominant among the Asians in the included studies, which constitutes a lack of ethnic diversity. Finally, we could not assess gene-to-gene and gene-to-environment interactions.

## Conclusion

In conclusion, our meta-analysis demonstrated that *SCN1A* rs2298771 polymorphism was significantly associated with resistance to AEDs in overall population and Caucasians (South Asians). However, we could not identify significant correlations between *SCN1A* rs10188577, *SCN2A* rs17183814, and *SCN2A* rs2304016 polymorphisms and resistance to AEDs according to current evidence. Considering the limitations of the present study, further large-scale and multi-ethnic studies on this topic are needed to verify our findings.

## Data Availability Statement

The original contributions presented in the study are included in the article/supplementary materials, further inquiries can be directed to the corresponding author/s.

## Author Contributions

ML, RZ, and WL conceived, designed, and drafted the article. ML, RZ, and YL reviewed the literatures and extracted the data. QZ and YL analyzed and interpreted data and edited pictures. All authors contributed to the critical revision of the article.

## Conflict of Interest

The authors declare that the research was conducted in the absence of any commercial or financial relationships that could be construed as a potential conflict of interest.
